# Temporal changes in cell division rate and genotoxic stress tolerance in quiescent center cells of Arabidopsis primary root apical meristem

**DOI:** 10.1038/s41598-019-40383-2

**Published:** 2019-03-05

**Authors:** Rupak Timilsina, Jin Hee Kim, Hong Gil Nam, Hye Ryun Woo

**Affiliations:** 1 Center for Plant Aging Research, Institute for Basic Science (IBS), Daegu, 42988 Republic of Korea; 2 Department of New Biology, Daegu Gyeongbuk Institute of Science and Technology (DGIST), Daegu, 42988 Republic of Korea

## Abstract

Plant roots provide structural support and absorb nutrients and water; therefore, their proper development and function are critical for plant survival. Extensive studies on the early stage of ontogenesis of the primary root have revealed that the root apical meristem (RAM) undergoes dynamic structural and organizational changes during early germination. Quiescent center (QC) cells, a group of slowly dividing cells at the center of the stem-cell niche, are vital for proper function and maintenance of the RAM. However, temporal aspects of molecular and cellular changes in QC cells and their regulatory mechanisms have not been well studied. In the present study, we investigated temporal changes in QC cell size, expression of QC cell-specific markers (*WOX5* and *QC25*), and genotoxic tolerance and division rate of QC cells in the Arabidopsis primary root. Our data revealed the decreased size of QC cells and the decreased expression of the QC cell-specific markers with root age. We also found that QC cell division frequency increased with root age. Furthermore, our study provides evidence supporting the link between the transition of QC cells from a mitotically quiescent state to the frequently dividing state and the decrease in tolerance to genotoxic stress.

## Introduction

Roots are vital plant organs that provide an anchorage for the plant’s aerial parts, as well as absorb nutrients and water; hence, the proper development and function of roots are essential for plant survival^1–3^. Extensive examinations of the roots of diverse plant species, such as *Arabidopsis*, maize, and rice, have revealed the organization of tissues and their distinct features during early root development^1,4–6^. The Arabidopsis primary root, one of the well-studied root systems, can be categorized into three zones: meristematic, elongation, and differentiation zones^1,7,8^. The cell division zone at the root tip is defined as the root apical meristem (RAM), which serves as a constant source of cells for the growth and development of the root system^1,7,9,10^. The RAM comprises the stem cell niche (SCN), containing pluripotent stem cells capable of undergoing multiple rounds of cell division without differentiation^7,9,10^. Above the SCN, there is a large population of actively dividing cells that gradually differentiate as they are displaced from the meristematic to the differentiation zone^1,4,7,10^. The cells in the SCN are sub-divided into mitotically inactive quiescent center (QC) cells and surrounding actively proliferating stem cell initials, namely, columella initials, cortex and endodermis initials, vascular initials, and stele initials^1,4,7,10–12^. In this respect, QC cells function as an organizing center to maintain the neighboring active stem cell initials^13^. Auxin is the primary driving force for QC establishment and specification^1,11^. Through the coordinated actions of various auxin transporters, an auxin maximum is established at the RAM, thereby specifying QC cell identity^11,14,15^. QC cells can also serve as a pool of backup cells for the replenishment of dead and/or damaged stem cell initials through the generation of new initials that push the dead cells out of the SCN^11,16^. It is noteworthy that QC cells are highly resistant to various DNA damaging agents, such as bleomycin, UV-B radiation, and X-rays, whereas actively dividing meristematic cells and stem cell initials are highly susceptible^11,16–18^. Taken together, studies examining QC cells indicate that they are critical for the development and maintenance of the RAM.

A defining feature of QC cells is mitotic quiescence^7,11,12,16^. Indeed, QC cells were initially identified as a group of cells with relatively low mitotic activity that are localized at the convergence of different root cell files^11,16^. Many studies have been dedicated to revealing the regulatory mechanisms underlying the mitotic quiescence of QC cells. One of the principal regulators that control the proliferation rate of QC cells is the WUSCHEL-RELATED HOMEOBOX 5 (WOX5) transcription factor, whose transcripts are exclusively expressed in QC cells^1,11,19^. WOX5 is a repressor of QC cell division, and loss of *WOX5* function results in the enlargement of QC cells^11,19^. Moreover, targeted protein degradation appears to be essential for controlling QC cell division. A study of the CELL CYCLE SWITCH 52 (CCS52) proteins, which are activators of the highly conserved Anaphase Promoting Complex/Cyclosome (APC/C), demonstrated the necessity of APC/C activity to maintain the quiescence of the QC cells^20^. ETHYLENE RESPONSE FACTOR 115, the rate-limiting factor for QC cell division, was identified as an APC/C^CCS52A2^ target for proteasomal degradation^21^. Nevertheless, information regarding temporal aspects of the regulatory mechanisms contributing to the mitotic quiescence of QC cells is very limited.

Under normal conditions, the cell cycle length of the QC cells in *Arabidopsis* exceeds 3 days^11,12,16,17,22^, three- to six-fold longer than that of its surrounding stem cell initials^23^. However, the proliferation rate of QC cells can be enhanced under specific stress conditions, such as elevated temperature or genotoxin treatments^16,24^. For example, treatment with hydroxyurea, a ribonucleotide reductase inhibitor that delays S-phase entry, significantly increases the frequency of QC cell division^16^. Increased levels of plant hormones, such as ethylene, jasmonic acid, and brassinosteroids, also facilitate QC cell division by transmitting a stress response signal^11,22,25–29^. In addition, cytokinins promote QC cell division by downregulating the expression of several key regulatory genes in the root tip, including *SCARECROW* (*SCR*), *WOX5*, and the auxin influx carrier genes (*AUXIN TRANSPORTER PROTEIN 1* and *LIKE AUXIN RESISTANT 2*)^30^. However, temporal alterations in the properties of QC cell division in response to these stresses or hormones have not been investigated in detail.

Since the vast majority of studies involving the QC cells in *Arabidopsis* have been focused on a particular time window of early root development, usually from 4 to 7 days after germination^12,13,16,18,30^, our knowledge of the regulatory mechanisms underlying the establishment and maintenance of the QC cells as the root ages is still fragmentary. In the present study, we performed temporal analysis of cell size, expression of QC cell-specific markers as well as genotoxic tolerance and division rate of QC cells, in the Arabidopsis primary root. Our data revealed dynamic temporal changes in size and regulatory gene expressions and an inverse correlation between the division rate and the tolerance to genotoxic stress of QC cells.

## Results

### Size of QC cells and expression of QC cell-specific marker genes in the primary RAM are temporally changed

**Figure 1:**
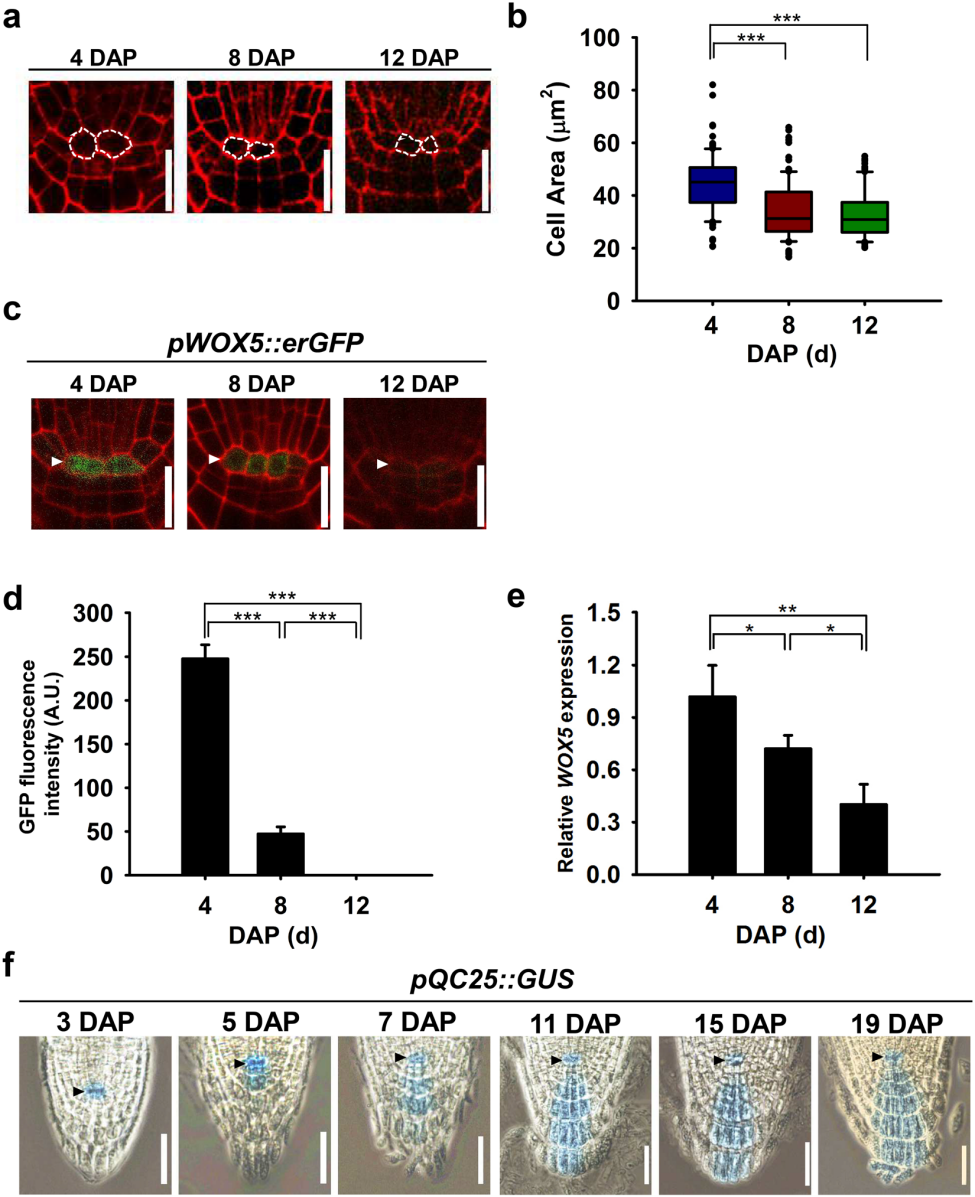
Temporal changes in size of quiescent cell (QC) cells and expression of QC cell-specific markers. (**a**) Representative confocal images of PI-stained stained root apical meristem (RAM) at 4 (left), 8 (middle), and 12 DAP (right). The QC cells are outlined with dashed lines. Scale bars, 20 μm. (**b**) Box and whisker plots showing the distribution of QC cell area at 4, 8, and 12 DAP (*n* = 100 for each time points). Boxes represent interquartile range while whiskers and dots represent total range. Statistical analysis was performed using two-tailed Student’s *t*-test (***p < 0.001). (**c**) Representative confocal images of PI-stained primary root tips expressing *pWOX5::erGFP* at 4, 8, and 12 DAP. Scale bar, 20 μm. (**d**) Quantification of pWOX5::erGFP fluorescence from (**c**) via image analysis of confocal sections. Data represent means ± SD (*n* = 15) from three independent trials. Statistical analysis was performed using two-tailed Student’s *t*-test (***p < 0.001). (**e**) Relative expression levels of *WOX5* at 4, 8, and 12 DAP. The *WOX5* transcript level was analyzed by RT-qPCR, normalized to *ACT2*, and shown as relative values to the level at 4 DAP. Data represent means ± SD (*n* = 3). Statistical analysis was performed using two-tailed Student’s *t*-test (*p < 0.05; **p < 0.005). (**f**) GUS expression driven by the *QC25* promoter in the primary RAMs at the number of days indicated. White and black arrowheads indicate the QC cells in (**c**,**f**), respectively. DAP, days after planting; Scale bar, 50 μm.

Cell size is an emergent property controlled by various factors such as frequency of cell division, intrinsic and extrinsic environmental cues, and developmental stage^31–33^. As the first step to characterize temporal changes in the properties of QC cells, we examined size of QC cells at 4, 8, and 12 days after planting (DAP). Size of QC cells at 4 DAP was significantly larger than those at 8 and 12 DAP (Fig. 1a,b, Supplementary Fig. 1). Mean cell area at 4, 8, and 12 DAP was 44.8, 34.2, and 32.7 μm^2^, respectively (Supplementary Fig. 1b). Likewise, mean length of QC cells at 4 DAP (9.4 μm) was significantly longer than those at 8 DAP (7.8 μm) and 12 DAP (7.3 μm), while the differences in mean height of QC cells at the examined time points were not significant (Supplementary Fig. 1c,d).

To investigate temporal dynamics of the regulatory mechanisms underlying the establishment and maintenance of the QC cells, we then examined molecular changes within the QC cells using well-characterized QC cell-specific marker lines: *pWOX5::erGFP* (gene encoding for endoplasmic reticulum localized GREEN FLUORESCENT PROTEIN under control of the *WOX5* promoter)^34^ and *pQC25::GUS* (gene encoding for β*-*GLUCURONIDASE under control of the *QC25* promoter)^35^ reporter lines. As expected, a strong pWOX5::erGFP signal was observed, particularly in the QC cells, at 4 DAP, but the GFP signal rapidly declined from 4 to 12 DAP (Fig. 1c). As shown in Fig. 1d, the relative GFP fluorescence intensity from the QC cells at 4 DAP was 247.7 units, but the signal dropped sharply at 8 DAP and was almost undetectable by 12 DAP. This observation was verified by reverse transcription quantitative polymerase chain reaction (RT-qPCR) analysis of the *WOX5* transcript level; it decreased to 69.0% at 8 DAP and 39.4% at 12 DAP, compared with 4 DAP (Fig. 1e). This time-dependent decrease of the *WOX5* expression implies the dwindled importance of WOX5 in QC cells during primary root development. In contrast, the expression of the QC cell-specific marker *QC25* was found to be sustained throughout the examined period in the *pQC25::GUS* transgenic line (Fig. 1f). Unexpectedly, GUS activity was also detected in columella cell layers from 7 DAP onwards (Fig. 1f), which suggests that *pQC25::GUS* expression is QC cell-specific only during the early stage of primary root growth. We, next investigated temporal changes in the activity of a synthetic auxin-responsive promoter *DR5*_*rev*_ which is expressed in QC cells^36^. Strong pDR5_rev_::GFP activity was observed in the QC cells, vascular initials, columella initials, and matured columella cell layers at 4 DAP, but it gradually decreased with root age (Supplementary Fig. 2). Taken together, these results indicate that the QC cells in Arabidopsis primary roots undergo dynamic temporal changes in cell size and regulatory gene expression.

### Genotoxic stress tolerance of QC cells decreases with root age

**Figure 2:**
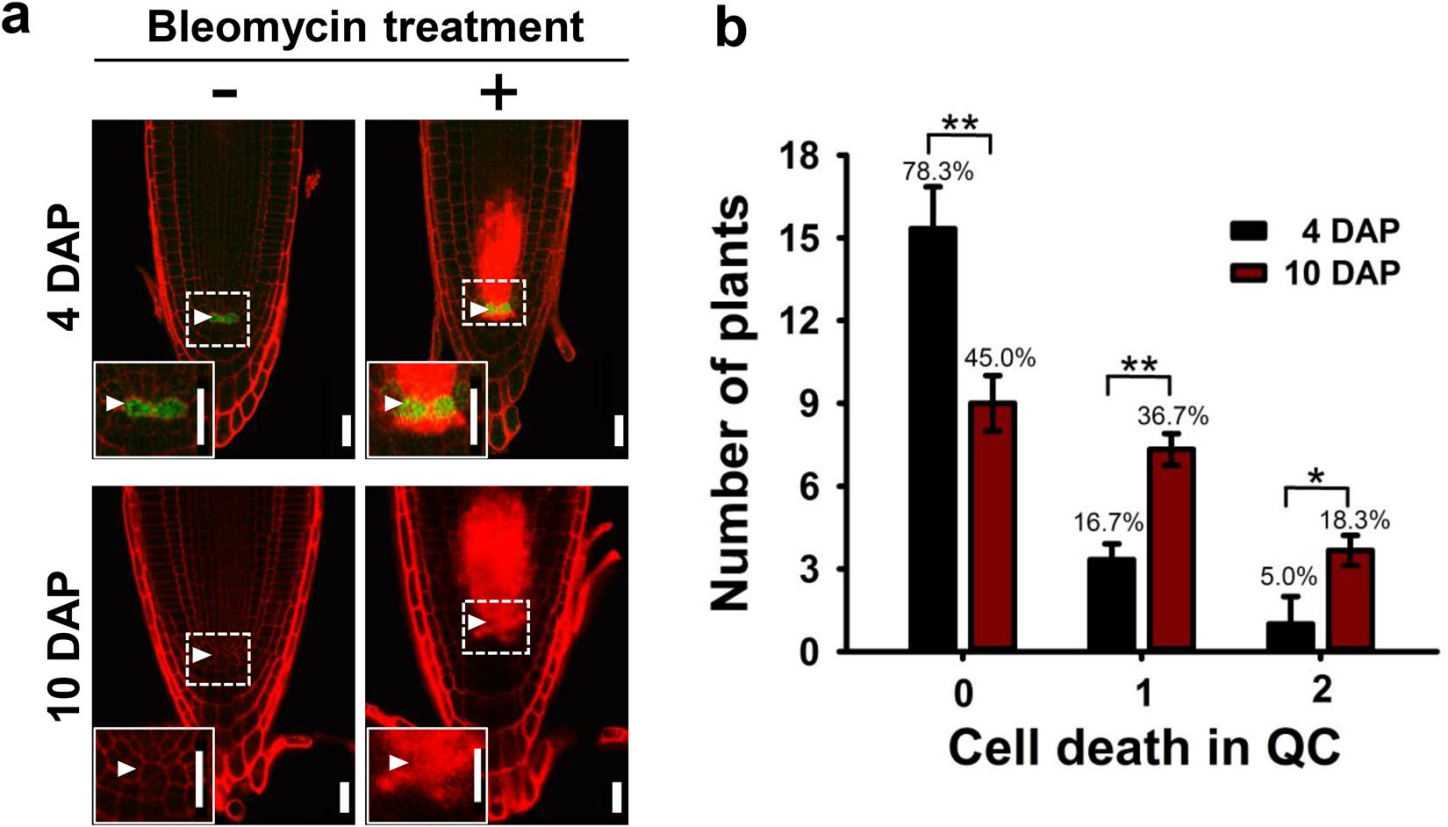
Comparison of the genotoxic effects of bleomycin on the QC cells in seedlings at 4 and 10 DAP. (**a**) Representative confocal images of the *pWOX5::erGFP* primary roots with (right column) or without (left column) 1 μg/ml bleomycin treatment for 1 day at 4 DAP (upper row) and 10 DAP (lower row). PI-stained cell walls indicate living cells, whereas intense red PI staining inside cells indicates dead cells. The insets at the bottom left show close-ups of the QC cells. White arrowheads indicate the QC cells. DAP, days after planting; Scale bar, 20 μm. (**b**) The number of plants harboring 0, 1, and 2 dead QC cells after bleomycin treatment and the respective percentages. Data represent means ± SD (*n* = 20) from three independent trials. Statistical analysis was performed using two-tailed Student’s *t*-test (*p < 0.05; **p < 0.01).

One distinct characteristic of QC cells is their extreme tolerance to various kinds of stress that can cause damage, and even death, of other types of cells in RAMs^12,16–18^. However, it was not clear whether QC cells can maintain such tolerance as they age. We thus performed a comparative study of genotoxic stress (treatment of 1 μg/ml bleomycin, a chemical that causes double-stranded DNA breaks^17,37^, for 1 day) response in the QC cells of the *pWOX5::erGFP* transgenic seedlings at 4 and 10 DAP. Consistent with the results of previous reports^12,16,18^, the QC cells of the seedlings at 4 DAP remained alive and intact upon bleomycin treatment, whereas other stem cell initials were susceptible [cells were stained with propidium iodide (PI), a marker for membrane integrity loss and cell death^38^; see Fig. 2a, upper right]. Unexpectedly, we encountered frequent incidents of QC cell death upon bleomycin treatment in the seedlings at 10 DAP (Fig. 2a, lower right). We then categorized the QC cell death incidents as 0, 1, and 2 QC deaths; in the 4-DAP seedlings, on average (3 independent trials with 20 samples each) 15.7 of 20 (78.3%) roots exhibited no QC cell death (0 QC death), 3.3 (16.7%) roots exhibited 1 QC cell death, and 1.0 (5.0%) roots exhibited 2 QC cell deaths. In contrast, in the 10-DAP seedlings, an average of 9.0 (45.0%), 7.3 (36.7%), and 3.7 (18.3%) roots exhibited 0, 1, and 2 QC deaths, respectively (Fig. 2b). These data indicate that the QC cells in the older RAMs were less tolerant to bleomycin treatment than those in the younger RAMs.

### QC cells protect themselves from genotoxic stress via mechanisms other than asymmetric chromosomal segregation (ACS)

**Figure 3:**
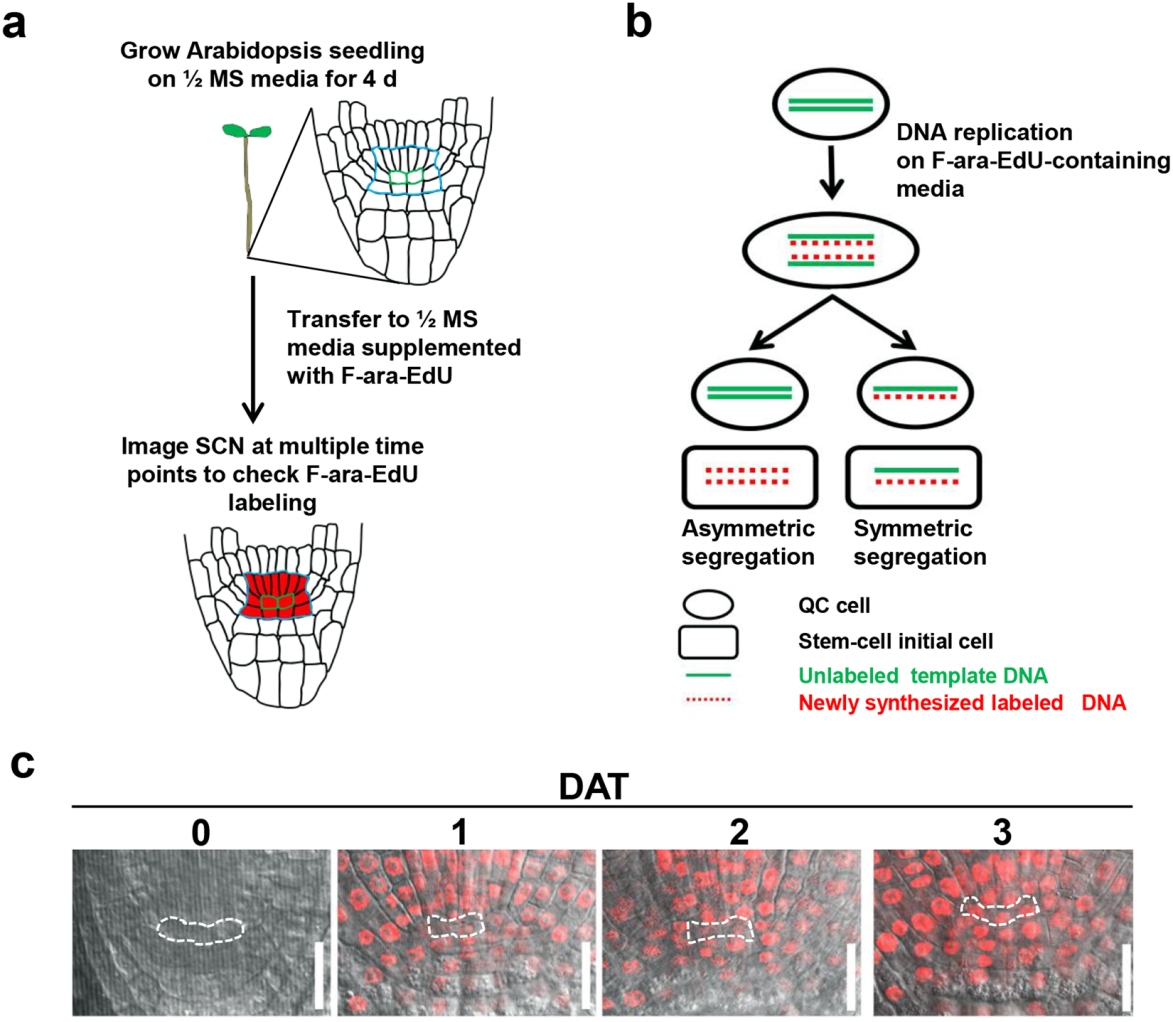
F-ara-EdU labeling patterns of the SCN in Arabidopsis primary roots. (**a**) Outline of the experimental strategy testing whether asymmetric chromosomal segregation (ACS) occurs during QC cell division. SCN, blue outline; QC, green outline. (**b**) Theoretical illustration of symmetric or ACS upon QC cell division in F-ara-EdU-supplemented media. (**c)** Representative confocal image of F-ara-EdU labeling of the SCN in 4-DAP seedling roots before transfer (0 DAT) and after 1–3 DAT from 1/2 MS media to F-ara-EdU-supplemented media. Red fluorescence indicates incorporation of F-ara-EdU into newly synthesized DNA during cell division. The QC cells are outlined with dashed lines. DAP, days after planting; DAT, days after transfer; Scale bars, 20 μm.

As shown in Supplementary Fig. 3, despite decrease in the tolerance to genotoxic stress shown by the older QC cells, they were still more tolerant to bleomycin treatment than the columella initials. Thus, we further investigated the possible mechanisms underlying such resistance to cell death in the QC cells. It has been suggested that ACS, the phenomenon of selective/non-random retention of parental DNA strands in one of the dividing daughter cells, is vital for maintaining certain types of stem cells in animals by protecting against the inheritance of DNA replication errors, preventing loss of telomeres during division, or preserving the epigenetic features of the stem cells^39,40^. We designed an experiment to determine whether ACS is an underlying mechanism for genotoxic stress resistance of the QC cells using (2′S)-2′-deoxy-2′-fluoro-5-ethynyluridine (F-ara-EdU), a metabolic label for newly synthesized DNA^41^ (Fig. 3a); if ACS occurred during QC cell division, we expected that the DNA in the QC cells would remain unlabeled (Fig. 3b). The Arabidopsis seedlings at 4 DAP were transferred to 1/2 Murashige and Skoog (MS) media supplemented with F-ara-EdU, and the incorporation of F-ara-EdU label into the RAM cells was monitored, particularly in the QC cells, from 1 to 3 days after transfer (DAT). At 1 DAT, most of the RAM cells were labeled, whereas the QC cells were rarely labeled (Fig. 3c). One or two QC cells in all examined RAMs, visible in the plane of confocal image, were labeled at 2 DAT, and all QC cells were labeled at 3 DAT (Fig. 3c). These results suggest that ACS does not occur during QC cell division.

Given that specific environmental stress conditions can facilitate the asymmetric segregation of damaged macromolecules, including DNA in a variety of organisms^42–45^, we examined whether genotoxic, salt, or osmotic stress causes ACS in the QC cells of Arabidopsis primary roots. Arabidopsis seedlings at 4 DAP were transferred to media containing 0.2 μg/ml bleomycin, 50 mM NaCl, or 50 mM mannitol. At 3 DAT, most of the QC cells were labeled with F-ara-EdU (Supplementary Fig. 4), indicating no occurrence of ACS even under such stress conditions. We then examined the possibility that the genes involved in DNA damage response and repair, such as *ATAXIA TELANGIECTASIA MUTATED (ATM)*, *ATM AND RAD3-RELATED* (*ATR*)^17^, and *SUPPRESSOR OF GAMMA RESPONSE 1 (SOG1)*^46^, regulate chromosomal segregation during QC cell division because the stem cell initials carrying loss-of-function mutations in these genes were reported to be resistant to cell death upon genotoxin treatment. As shown in Supplementary Fig. 5, we found no occurrence of ACS in the *atm-2*, *atr-2*, or *sog1-1* mutants. Collectively, our results suggest that QC cells in the RAM are protected from genotoxic stress via mechanisms other than ACS.

### The rate of QC cell division is temporally regulated

**Figure 4:**
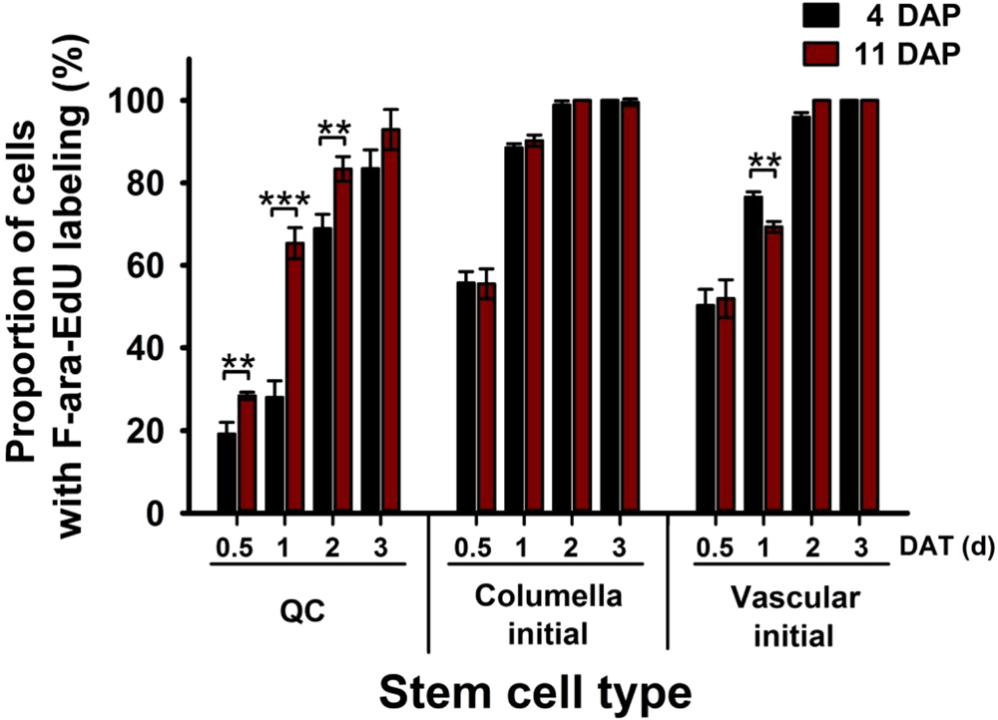
Comparison of cell division rates of various stem cell types in seedlings at 4 and 11 DAP. The graph shows the cell division rates in the QC (left), columella initial (middle), and vascular initial (right) cells. Entry into the S-phase of the cell cycle was monitored in the RAM of primary roots of 4- (black bars) and 11-DAP (red bars) seedlings at 0.5, 1, 2, and 3 DAT from 1/2 MS media to F-ara-EdU-supplemented media. Cells entering the S-phase were determined by red fluorescence of Alexa 555 dye conjugated with F-ara-EdU, which is incorporated into newly synthesized DNA during cell division. Data represent means ± SD (*n* = 15) from three independent trials. Statistical analysis was performed using two-tailed Student’s *t*-test (**p < 0.01; ***p < 0.001). DAP, days after planting; DAT, days after transfer.

Since actively dividing stem cells are more sensitive to genotoxic stress than slowly dividing cells^16,17,47^, we hypothesized that the increased susceptibility of the QC cells to genotoxic stress with increasing age may be due to their compromised mitotic quiescence. To test this, we measured the rate of QC cell division by employing the F-ara-EdU DNA labeling strategy. Arabidopsis seedlings at 4 and 11 DAP were transferred to F-ara-EdU-supplemented media, and the fraction of cells that entered the S-phase of the cell cycle, at 0.5, 1, 2, and 3 DAT, was quantified. As reported previously^12,16^, the QC cells of the 4-DAP seedlings divided much more slowly than columella and vascular initial cells (Fig. 4 and Supplementary Fig. 6). Furthermore, we observed that the fractions of dividing QC cells in the 11-DAP seedlings were significantly higher than those in the 4-DAP seedlings, 1.48-fold (p < 0.05), 2.33-fold (p < 0.001), and 1.21-fold (p < 0.05) higher at 0.5, 1, and 2 DAT, respectively (Fig. 4). The division rate of the QC cells in the 11-DAP seedlings was comparable to that of vascular initial cells at 1 DAT (Fig. 4). These results suggest that root age is a critical factor that transitions QC cells from a mitotically quiescent state to an actively proliferating state.

To investigate possible molecular mechanisms underlying the increased QC cell division with root age, we examined changes in the expression of *CyclinB1;1* (*CYCB1;1*), a cell proliferation marker, in the primary root of the Arabidopsis *pCYCB1;1::GUS* reporter line^48^. We observed that only 22.5% (10 out of 44) of the samples at 4 DAP exhibited GUS activity in QC cells. In contrast, the majority of the samples i.e. 51.1% (23 out of 45), 51.6% (32 out of 62), and 72.5% (29 out of 40) at 8, 12, and 16 DAP, respectively, exhibited GUS activity in QC cells (Supplementary Fig. 7). It implies the possible involvement of cyclin-dependent pathways in the temporally increased division rate of the QC cells. Taken together, our results suggest that QC cells act as a reservoir of stem cell initials, accompanied by characteristically infrequent cell division and extreme tolerance to genotoxic stress, during the early stage of root development. However, QC cells become susceptible to genotoxic stress, likely because they transit from a quiescent state to a frequently dividing state, as do other stem cell initials.

## Discussion

Since the majority of the studies on roots has been limited to a specific time point in the early post-germination stage, knowledge of the molecular and cellular mechanisms governing the temporal dynamics of the RAM, especially QC cells, is very scarce. Herein, we elucidated dynamic changes in cell size and QC-specific marker gene expressions, as well as the differential features in the cell division rate and genotoxic stress response of the QC cells with root age. Our work highlights the temporal transition of QC cells from a quiescent state to a frequently dividing state.

In this study, we demonstrated temporal changes in size of QC cells (Fig. 1a,b, Supplementary Fig. 1). Optimal size of QC cells could be a critical factor to efficiently regulate juxtacrine signaling between QC cells and surrounding stem cell initials likely through modulation of contact surface area, number of plasmodesmata, and expression and localization of cell surface receptors and ligands^49^. Although the detailed mechanisms for size regulation of QC cells need to be elucidated, it is possibly related to temporal increase in the rate of QC cell division (Fig. 4). It appears that QC cells dynamically regulate their size to properly function in the SCN by controlling the balance between cell growth and division.

Our analysis further reveals that the expression of *WOX5*, a well-known QC cell marker gene, rapidly diminishes with root age (Fig. 1c–e). This is intriguing because WOX5 represses QC cell division and plays important roles in QC cell specification and establishment during embryogenesis of the primary root^1,11,19^. The significant decrease of *WOX5* expression in the QC cells with root age (Fig. 1c–e) implies that WOX5 may not be essential for QC cells once they are specified in the SCN. It is also feasible that the QC cells of old roots may no longer require maintenance at the mitotically quiescent state. Characterization of the changes in the QC cells by inducible overexpression or suppression of *WOX5* expression in aged roots might facilitate to dissect the roles of *WOX5* with root age. Alternatively, we cannot rule out the possibility that other regulators play the same functional role as WOX5 in aged QC cells. QC25 is also specifically expressed in QC cells^35^, but our work demonstrates that the expression of *QC25* reaches as far as the mature columella cell layers from 7 DAP onwards (Fig. 1f). While the exact role of QC25 has not been investigated, it appears that its function is limited to QC cells in the early stages of root development, but it functions in both QC cells and columella cells in later stages.

In our study, the rate of QC cell division increased observably with root age (Fig. 4). To the best of our knowledge, this is the first study to reveal the link between age and rate of QC cell division. Although the exact mechanism underlying the temporal loss of QC cell mitotic quiescence remains unclear, it may be explained in part by the decreased expression of the QC cell division repressor *WOX5* and the increased expression of *CYCB1;1* with increasing root age (Fig. 1c–e, Supplementary Fig. 7). *WOX5* is reported to suppress QC cell division by excluding *CYCD* activity from QC cells^50^. Therefore, further studies on spatial and temporal expression of cell cycle regulators such as *WOX5* and *CYCD* might provide some mechanistic insight into the increased rate of QC cell division with age. The repression of QC cell division by the action of WOX5 is regulated by retinoblastoma homolog (RBR) and a SCR heterocomplex. SCR, expressed in QC cells, functions as an upstream activator of *WOX5*^11,16,19^, whereas REPRESSOR OF WUSCHEL1, expressed in the cells above the QC cells (not in the QC cells themselves), functions as an upstream repressor of *WOX5*^51^. It is plausible that the altered expression of RBR and/or SCR in QC cells may lead to temporal decrease in *WOX5* expression. Further studies need to be conducted to determine the molecular networks that regulate QC cell division and whether these networks are strengthened or attenuated as the root ages.

We further provided the evidence to suggest that the increased QC cell division rate in old roots causes attenuated resistance to genotoxic stress. This is consistent with previous findings demonstrating that actively dividing stem cells are more sensitive to genotoxic stress than slowly dividing cells in both animals and plants^16–18,47^. We believe that the enhanced sensitivity of actively dividing QC cells to genotoxic stress may be because serious DNA damage in the dividing cells leads to the execution of cell death pathways, similar to those observed in animals^47^. Considering the enriched expression of genes involved in DNA repair in the QC cells compared with that in other root cells^52^, it is also possible that those genes are expressed at lower levels in actively dividing QC cells than in slowly dividing QC cells. The biological and physiological purpose of increasing the QC cell division rate at the cost of stress resistance raises intriguing questions. Considering that the plant root faces multiple biotic and abiotic stresses even under natural growth conditions and that several of these stresses affect stem cell maintenance^16,53^, an increase in QC cell division with root age may be a strategy to support and continually renew the SCN during the vigorously growing stage. This hypothesis could be tested utilizing clonal analysis tools such as the one in Heidstra *et al*.^54^ with a site-specific gene activation/deletion system in which induced clones are positively marked with GFP. Further identification of the molecular components that regulate age-dependent cell division and genotoxic tolerance of QC cells will advance our understanding of the molecular mechanisms underlying the temporal alterations in the maintenance and function of QC cells.

In conclusion, the present study reveals the temporal changes in cell size and regulatory gene expression in QC cells; we also observed the increase in QC cell division frequency with root age. Furthermore, we provide evidence implying that the temporal increase in susceptibility of QC cells to genotoxic stress may be due to their compromised mitotic quiescence.

## Methods

### Plant materials and growth conditions

The present study was conducted on *Arabidopsis thaliana*, ecotype Columbia (Col-0), and the transgenic lines *pWOX5::erGFP*^34^, *pQC25::GUS*^35^, *pDR5*_*rev*_*::GFP*^36^, *pCYCB1;1::GUS*^48^ have been described previously. Seeds were sterilized in 70% ethanol for 1 min, followed by 25% commercial bleach solution for 5 min, and washed several times with distilled water. Seeds were sown in 1/2 MS media (Duchefa, The Netherlands) containing 1% sucrose, 0.5 g/L 4-morpholineethanesulfonic acid (Amresco, USA), and 0.8% agar (pH 5.7). After stratification for at least 2 days at 4 °C in darkness, the seeds were sown in the media, and the plates were placed vertically under continuous 80 μmol m^−2^ s^−1^ light at 22 °C in an environmentally controlled growth room (Korea Instruments, Korea).

### Confocal microscopy, cell size measurement, and fluorescence intensity analysis

Confocal images of root z-stacks (2 μm sections) were obtained using a Zeiss LSM 7 DUO confocal laser scanning microscope (Carl Zeiss, Germany), with a 40× oil immersion objective. At least 5 seedlings from three independent experiments (total of 15 seedlings) were analyzed. Roots were stained with 10 μg/ml PI solution (Life Technologies, USA) for 1 to 10 min. Fluorescence intensity was acquired at 488/505–530 nm excitation and emission for GFP, 543/560 nm for PI, and 543/560–615 nm for Alexa Fluor 555. ZEN2012 software (Carl Zeiss, Germany), Image J (http://rsbweb.nih.gov/ij), and Photoshop were used to analyze the green fluorescence intensity. QC cell dimensions and area were measured using Image J. For measurement of QC cell dimensions, the longest sideward and upward distances between the plasma membrane were chosen as length and height, respectively. QC cell area was measured as area enclosed by free hand line drawn over the PI-stained plasma membrane of QC cells.

### RNA extraction and RT-qPCR analysis

RNA extraction and RT-qPCR were performed as reported^55^ with minor modifications. Total RNA was isolated from root tips (0.5 cm) at 4, 8, and 12-DAP seedlings using WelPrep^™^ RNA Isolation Reagent (Welgene, Korea). cDNA was synthesized using 1 µg total RNA using ImProm-II^TM^ system (Promega, USA) following the manufacturer’s instructions and used as template for qPCR (CFX96 system; Bio-Rad, USA) to determine transcript levels. Triplicate independent root samples were used for all analyses using the primers specific for *WOX5* (5′-GTG GCA ACA ATA ACG GAG G-3′ and 5′-TCT TGA CAA TCT TCT TCG CTT-3′) used previously^51^ and *ACTIN2* (*ACT2*) (5′-TCT TCC GCT CTT TCT TTC CAA GC-3′ and 5′-ACC ATT GTC ACA CAC ACG ATT GGT TG-3′).

### Histochemical analyses

Analysis of the GUS activity in primary root tips employed a histochemical assay, as described previously with minor modifications^56^. Seedlings or the excised roots of and *pCYCB1;1::GUS* and *pQC25::GUS* transgenic lines were immersed in GUS staining solution containing 2 mM 5-bromo-4-chloro-3-indolyl glucuronide, 2 mM K_3_Fe(CN)_6_, 2 mM K_4_Fe(CN)_6_, 10 mM EDTA, and 0.1 M PBS (pH 7.0) for 12 and 2.5 h at 37 °C for *pQC25::GUS* and *pCYCB1;1::GUS* lines, respectively; they were then destained with 70% (v/v) ethanol at room temperature for 2.5 h. The samples were then mounted on a chloral hydrate clearing solution (chloral hydrate:glycerol:water in a 8:3:1 ratio). Bright field photographs of the GUS-stained root samples were taken using a LEICA DFC450 C microscope (Leica Microsystems, Germany), with 40× objective.

### Chemical and stress treatments

Seedlings at 4 DAP were transferred to fresh 1/2 MS agar plates supplemented with 0.2 or 1 μg/ml bleomycin (24 h in dark), 75 mM NaCl, or 100 mM mannitol and vertically placed in continuous light at 22 °C in an environmentally controlled growth chamber for days mentioned in the main text.

### F-ara-EdU staining and imaging

F-ara-EdU labeling and detection was performed as previously described with minor modifications^57^. Seedlings grown in 1/2 MS were transferred to 1/2 MS media supplemented with 0.2 μM F-ara-EdU (Invitrogen Click-iT^®^ EdU Imaging Kit). Whole seedlings or excised roots were fixed in 3.7% (v/v) formaldehyde solution for 1 day, treated with permeabilization buffer [0.5% (v/v) Triton-X in 1× PBS] for 20 min, and washed twice in wash buffer (3% BSA in 1× PBS). Then, the samples were incubated in click-iT reaction mixture (Invitrogen Click-iT^®^ EdU Imaging Kit; 1× Click-iT^®^ EdU reaction buffer, CuSO_4_, Alexa Fluor^®^ azide, and 1× Click-iT^®^ EdU buffer additive) for 30 min and washed twice in wash buffer, followed by a final wash in 1× PBS buffer. All procedures were performed at room temperature. The samples were mounted on glass slides using 30% glycerol and imaged immediately using a Zeiss LSM 7 DUO confocal laser scanning microscope (Carl Zeiss, Germany), with a 40× oil immersion objective. At least 15 seedlings from three independent experiments were analyzed.

## Supplementary information

Supplementary text and figures

## Data Availability

The authors declare that all data supporting the findings of this study are available within the article and its Supplementary Information files.
