# Supplementary material for: Temporal changes in cell division rate and genotoxic stress tolerance in quiescent center cells of Arabidopsis primary root apical meristem
Source: Sci Rep. 2019 Mar 5;9:3599. doi: 10.1038/s41598-019-40383-2 (PMC6400898; doi:10.1038/s41598-019-40383-2)
Supplement: Supplementary file 1 — Supplementary text and figures [file 41598_2019_40383_MOESM1_ESM.pdf]

Temporal changes in cell division rate and genotoxic stress tolerance in quiescent center cells of Arabidopsis primary root apical meristem

Rupak Timilsina[1, 2], Jin Hee Kim[1], Hong Gil Nam[1, 2*] & Hye Ryun Woo[2*]

[1]Center for Plant Aging Research, Institute for Basic Science (IBS), Daegu 42988, Republic of Korea.

[2]Department of New Biology, Daegu Gyeongbuk Institute of Science and Technology (DGIST), Daegu 42988, Republic of Korea.

Rupak Timilsina and Jin Hee Kim contributed equally to this work.

Correspondence and requests for materials should be addressed to H.R.W. (email: hrwoo@dgist.ac.kr) or H.G.N. (email: nam@dgist.ac.kr).

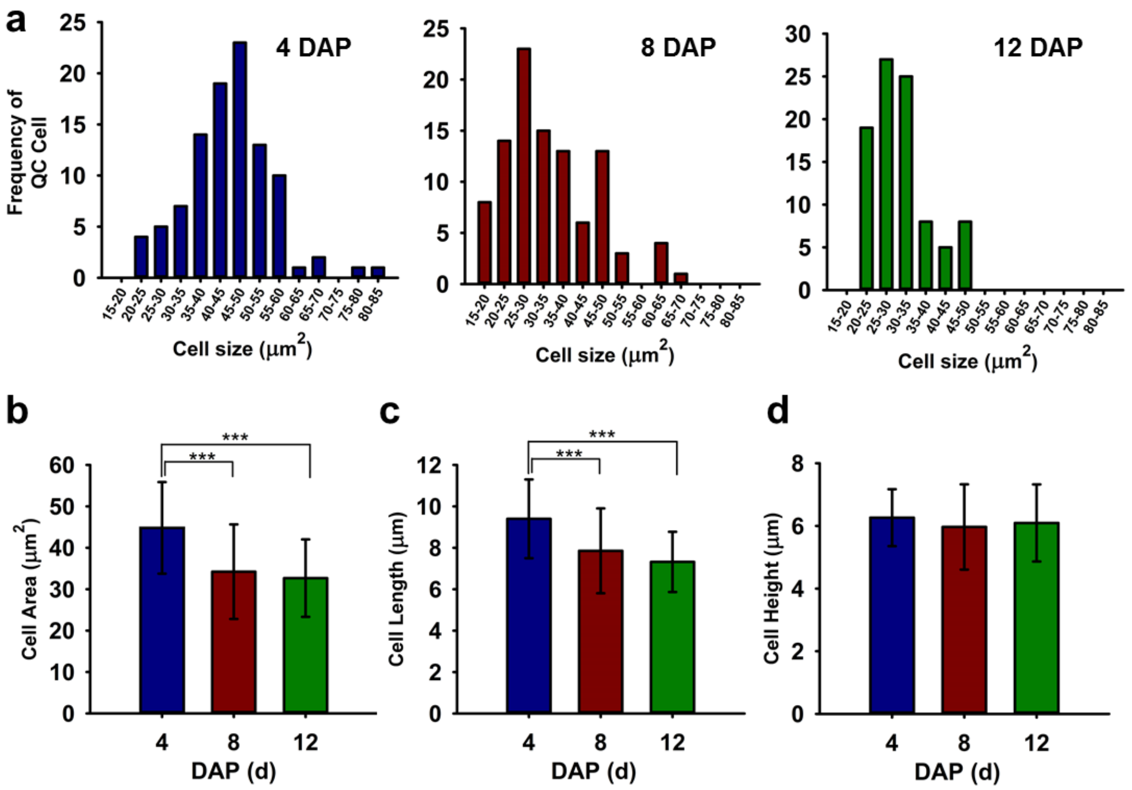

Supplementary Fig. 1. Temporal profiles of QC cell size. (**a**) Distribution of QC cell area at 4 (left), 8 (middle), and 12 DAP (right). (**b-d**) The average of area (**b**), length (**c**), and height (**d**) of QC cells at 4, 8, and 12 DAP, respectively. The total of 100 QC cells was analyzed in (**a-d**). Statistical analysis was performed using two-tailed Student's t-test (***, $p < 0.001$). DAP, days after planting.

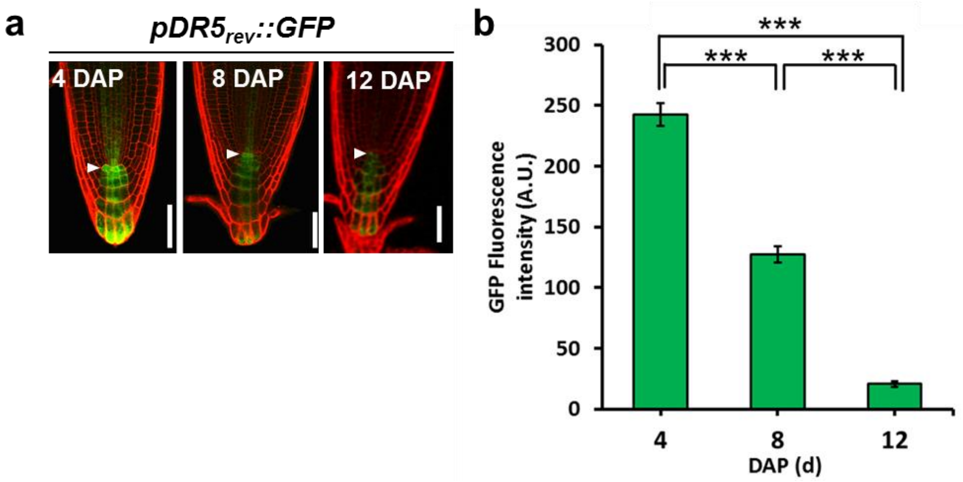

Supplementary Fig. 2. Temporal changes in expression of an auxin-responsive promoter *DR5$_{rev}$* in Arabidopsis primary RAM. (**a**) Representative confocal images of primary root tips expressing *pDR5$_{rev}$::GFP* at 4, 8, and 12 DAP. White arrowheads indicate the QC cells. DAP, days after planting; Scale bar, 50 µm. (**b**) Quantification of pDR5$_{rev}$::GFP fluorescence in QC cells from (**a**) via image analysis of confocal sections. Data represent means ± SD (*n* = 15) from three independent trials. Statistical analysis was performed using two-tailed Student's t-test (***, $p < 0.001$).

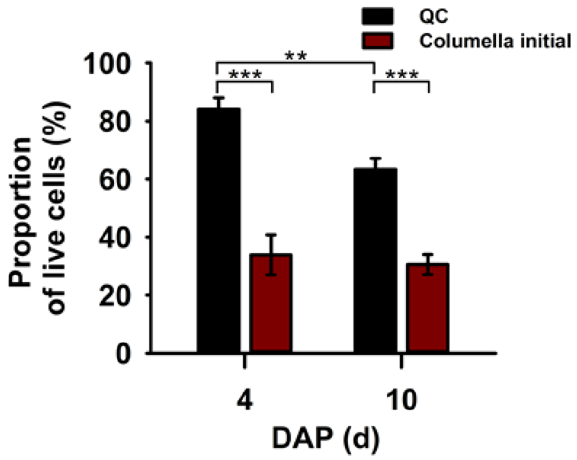

Supplementary Fig. 3. Comparison of genotoxin effects of bleomycin on the QC cells and columella initial cells at 4 and 10 DAP seedlings. The graph shows the percentage of alive QC cells and columella initial cells when 4- and 10-DAP root were treated with 1 µg/ml bleomycin for 1 day. . Data represent means ± SD (n = 20) from three independent trials (total of 60 samples). Statistical analysis was performed using two-tailed Student's t-test (**, $p < 0.01$; ***, $p < 0.001$). DAP, days after planting.

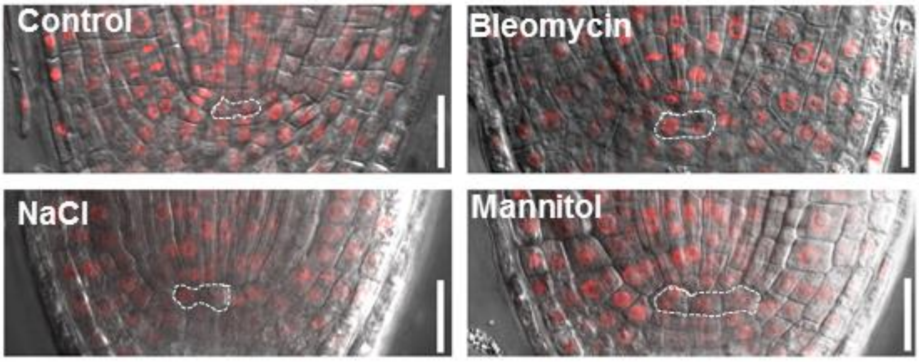

Supplementary Fig. 4. F-ara-EdU labeling patterns of SCN in Arabidopsis primary root under stress conditions. Representative confocal image of F-ara-EdU labeling of SCN in 4 DAP roots after 3 DAT to media containing F-ara-EdU and control (upper left), 0.2 μg/ml bleomycin (upper right), 50 mM NaCl (lower left), or 50 mM mannitol (lower right). Red fluorescence signals indicate incorporation of F-ara-EdU into newly synthesized DNA during cell division. The QC cells are outlined with dashed lines. DAP, days after planting; DAT, days after transfer; Scale bar, 20 μm.

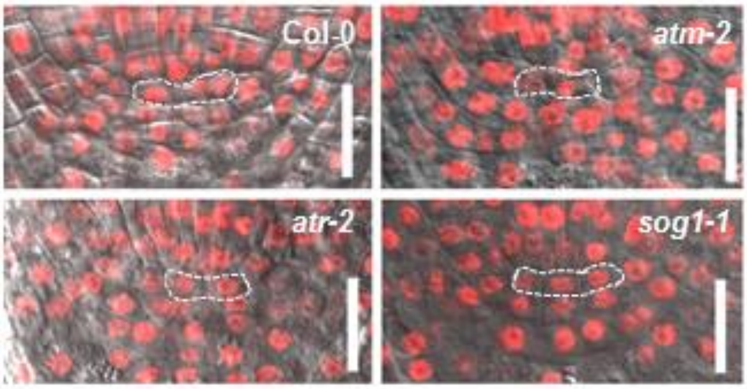

Supplementary Fig. 5. F-ara-EdU labeling patterns of SCN in Arabidopsis primary roots of DNA repair mutants. Representative confocal image of F-ara-EdU labeling of SCN in 4-DAP seedling roots of Col-0 (upper left), *atm-2* (upper right), *atr-2* (lower left), and *sog1-1* (lower right) after 3 DAT to media containing F-ara-EdU. Red fluorescence signals indicate incorporation of F-ara-EdU into newly synthesized DNA during cell division. The QC cells are outlined with dashed lines. DAP, days after planting; DAT, days after transfer; Scale bar, 20 μm.

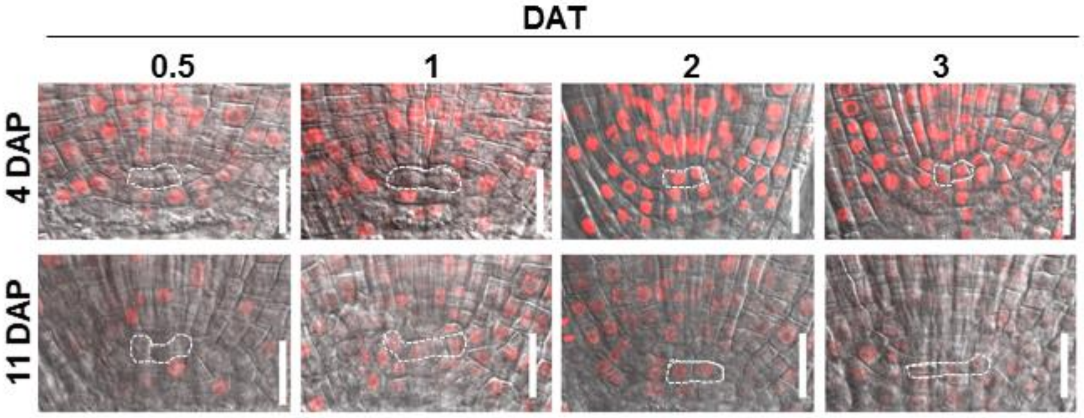

Supplementary Fig. 6. Representative confocal images of SCN in 4- and 11-DAP seedlings transferred from ½ MS media to F-ara-EdU supplemented media. Representative confocal image of F-ara-EdU labeling of DNA of cells in SCN after transfer of 4- (upper rows) and 11- (lower row) DAP seedlings from 1/2 MS media to F-ara-EdU supplemented media for indicated days. Red fluorescence signals indicate incorporation of F-ara-EdU into newly synthesized DNA during cell division. The QC cells are outlined with dashed lines. DAP, days after planting; DAT, days after transfer; Scale bar, 20 μm.

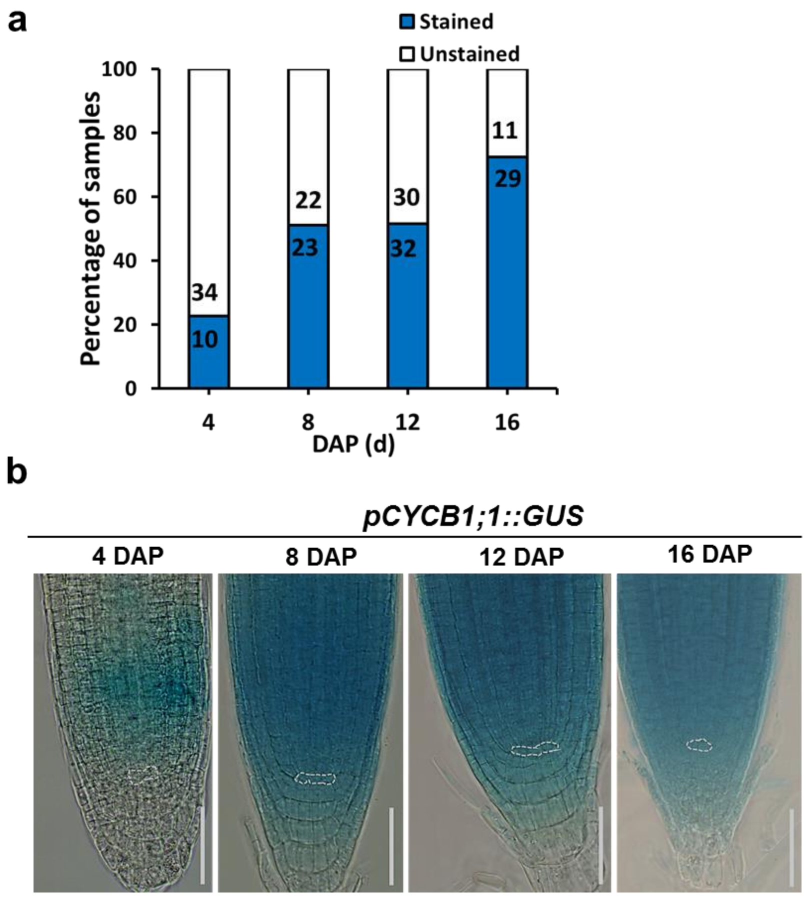

Supplementary Fig. 7. Temporal changes in expression of a cell division marker in QC cells. (**a**) The graph showing the proportions of samples with GUS activity in QC cells at the number of days indicated. The numbers inside each bar denote the number of samples with or without GUS activity in QC cells. DAP, days after planting. (**b**) Representative root images showing GUS activity of *pCYCB1;1::GUS* seedlings from 4 to 16 DAP. The QC cells are outlined with dashed lines. DAP, days after planting; Scale bars, 50 µm.
